# A Scientific Poultice

**Published:** 1907-07-20

**Authors:** 


### A SCIENTIFIC POULTICE.

The action of hot poultices is three-fold. They protect inflamed parts from further infection or injury; they cause an active hyperæmia of diseased structures and of the healthy tissues immediately round about them; and, in the case of antiseptic poultices, they inhibit the multiplication of the micro-organisms with which the fluid parts of the poultice are able to come in contact.

The last-mentioned action is wanting if there is no antiseptic present; for example, in a hot-water poultice, a bread poultice, or a linseed poultice. It is on this account that boric acid fomentations are more efficacious than are simple hot-water poultices; nevertheless, many of the non-antiseptic poultices do so much good that one realises that the main service of the application is to draw blood to the part by the use of continuous, circumscribed, and enclosed damp heat. The gutta-percha tissue is essential to efficient action, not merely because it keeps in the moisture, but also because it delays the cooling of the poultice.

### How it Acts.

It is probable that the local active hyperæmia which results from poulticing is beneficial in two ways at least; first, the deleterious products are removed from the diseased part, to be dealt with and destroyed elsewhere by healthy body cells; secondly, the number of exuded leucocytes is greatly increased, the army of defence is augmented and becomes one of offence against the invading micro-organisms. The latter factor is most important in the cure; leucocytes which are destroyed by bacteria accumulate as pus, and it is because of the greater number of leucocytes drawn to the affected part by the poultices that abscesses are hastened, and the inflammation is "brought to a head."

### Its Effects.

The question arises: Is there any means of increasing this mode of action of a poultice? One would naturally suppose that poultice to be most efficient which causes the greatest determination of active leucocytes to the part. Most poultices do so merely by keeping the affected region constantly hotter and damper than the surrounding parts, and they therefore differ but little amongst themselves as regards efficiency in this respect. It is possible, however, to bring about a still more active determination of leucocytes to the part if the poultice is not only hot and moist but also contains a substance of high osmotic power. Osmosis can have but little influence through an unbroken skin, it is true; it is particularly when the surface to which the application is to be made is broken—a whitlow that has been opened, for example, or a discharging carbuncle—that a poultice which contains a substance of high osmotic power will be of value. The simplest substance for the purpose is *cane-sugar*. This has a high osmotic power, attracting much lymph to the broken surface to which it is applied; it causes no pain to the patient; it is cheap; it is always at hand. For broken surfaces, therefore, a 10 per cent. solution of cane-sugar is a much more efficient basis for a poultice than is plain water. There is no reason, of course, why an antiseptic such as boric acid should not be used in the same solution.

### The Poultice Itself.

It often happens that the effects of poultices are considerably interfered with by scabs or clots covering the affected area. Leucocytes are free to move about in warm fluids by amœboid movements, and the more fluid the discharge from a septic focus, the more freely can leucocytes get from place to place to attack invading micro-organisms. The clotting or coagulation of blood-plasma and of its exudates interferes with the amœboid movements of the leucocytes, and any procedure which prevents the formation of a coagulum at the same time hastens the cure of the disease. The best-known inhibitor of blood-coagulation is potassium citrate; it seems a reasonable suggestion, therefore, that poultices for broken surfaces should contain small quantities of this salt. It will not poison the patient; it will not pain him if it is used in weak solution, such as 1 per cent.; and it will not do any damage locally. The ideal lotion for making a poultice for a suppurative lesion with a broken surface will therefore be:

| | |
|---|---|
| Potassii citratis ... ... ... | gr. xcvi. |
| Sacchari communis ... ... ... | ℥ij. |
| Aquam ad ... ... ... ... | Oj. |

or

| | |
|---|---|
| Potassium citrate ... ... ... | 1 part |
| Ordinary sugar ... ... ... | 10 parts |
| Water to ... ... ... ... | 100 parts |

### How to Use It.

The poultice is used precisely as other poultices are; the lotion is boiled; lint in a wringer is wrung out of it not too dry, applied as hot as the patient can bear it to the affected part, quickly covered with gutta-percha tissue and cotton wool which are kept on with a bandage. The application should be renewed every four hours, for its delicacy is greatly diminished when the poultice has got cold; nevertheless a cold potassium citrate and sugar poultice is not so inefficient as a cold boric acid poultice, so that when for any reason there is a difficulty in getting the application renewed at the required intervals there is an additional advantage in using the former. Boric acid may be dissolved in the lotion, to the point of saturation, if an antiseptic action is required; or, especially for lesions in the region of the perinæum or other site where the bacillus coli communis is the offending organism, creolin is to be preferred. Creolin has a wonderful power of making sweet such foci as have been infected with bacillus coli.

### Its Use in Furunculosis.

The potassium citrate and sugar poultices are most efficient in cases of carbuncles and huge boils. An incision is first required, and then the poultices, by their combined actions of preventing coagulation and of attracting lymph by osmosis, cause a most abundant discharge for the first day or two, and the boil or carbuncle is usually well in a day or two after that.

